# Supplementary material for: Src activates retrograde membrane traffic through phosphorylation of GBF1
Source: eLife. 2021 Dec 6;10:e68678. doi: 10.7554/eLife.68678 (PMC8727025; doi:10.7554/eLife.68678)
Supplement: Source data 1. — The red boxes indicate the regions of the blot presented in the figures of the article. [file elife-68678-supp1.pdf]

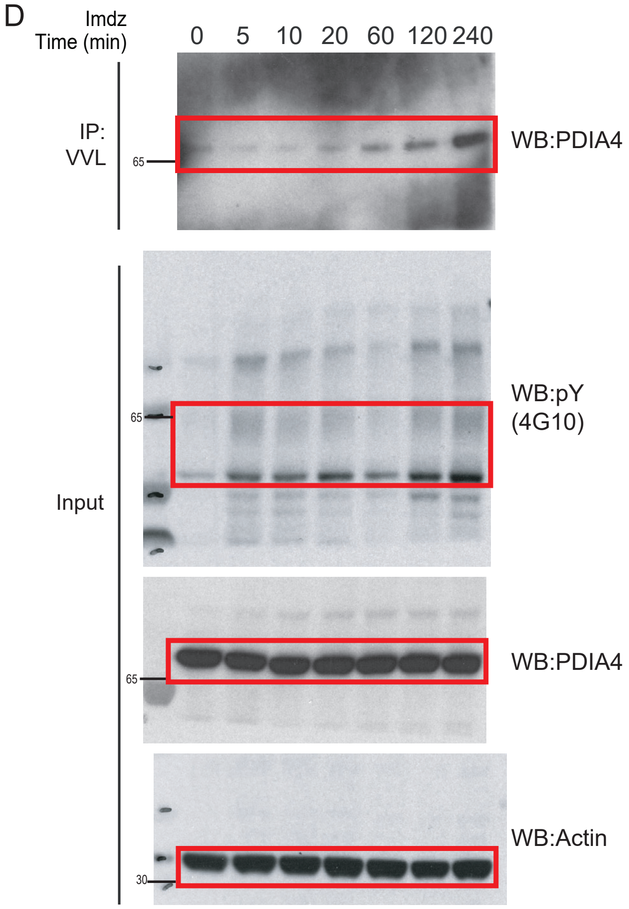
D
Imdz
Time (min)
0
5
10
20
60
120
240
IP:
VVL
65
WB:PDIA4
Input
65
WB:pY
(4G10)
65
WB:PDIA4
WB:Actin
30

Figure 1

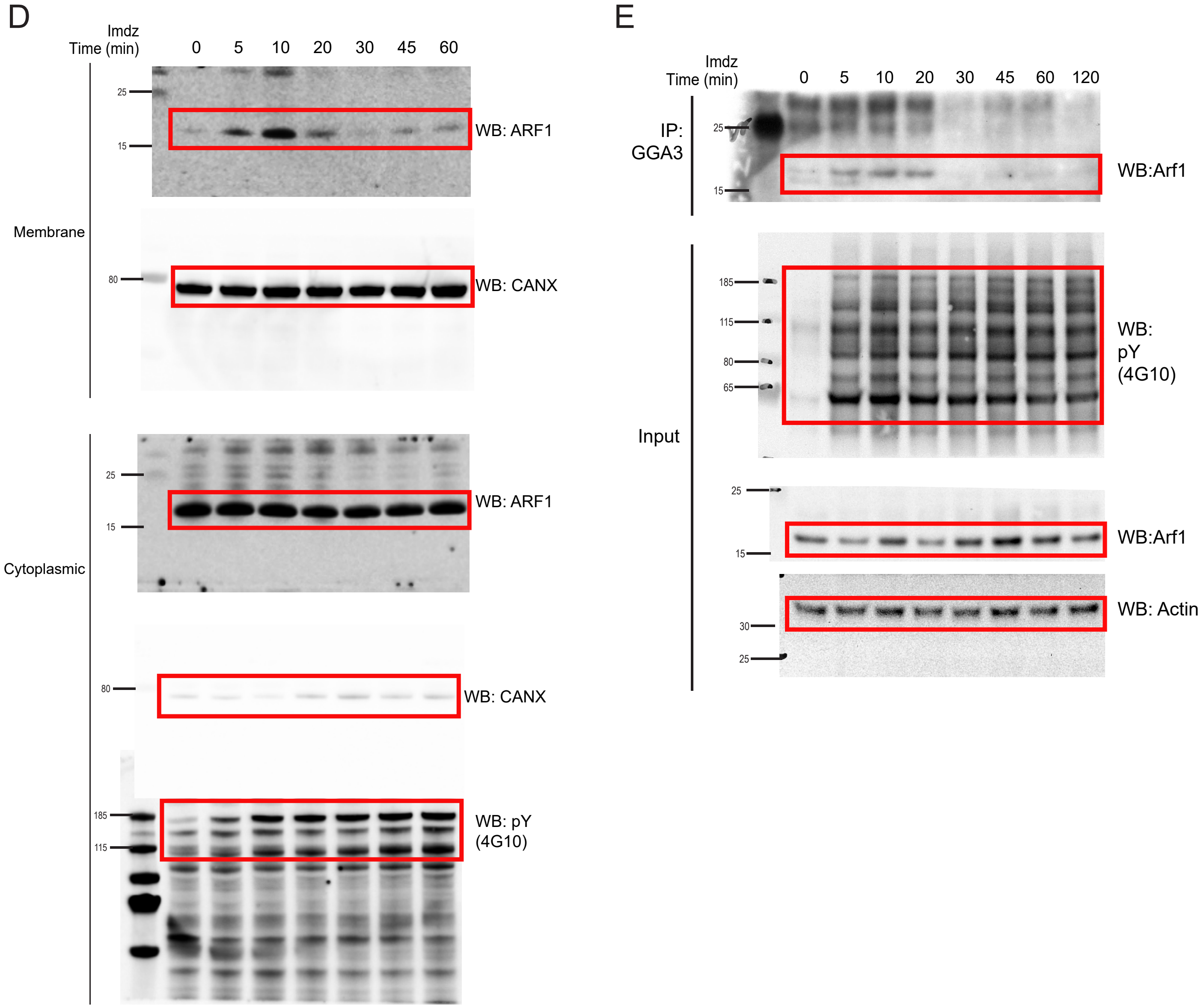

D
Imdz
Time (min)
0 5 10 20 30 45 60
25
15
WB: ARF1
Membrane
80
WB: CANX
25
15
WB: ARF1
Cytoplasmic
80
WB: CANX
185
115
WB: pY (4G10)
E
Imdz
Time (min)
0 5 10 20 30 45 60 120
IP: GGA3
25
15
WB:Arf1
Input
185
115
80
65
WB: pY (4G10)
25
15
WB:Arf1
30
25
WB: Actin

Figure 2

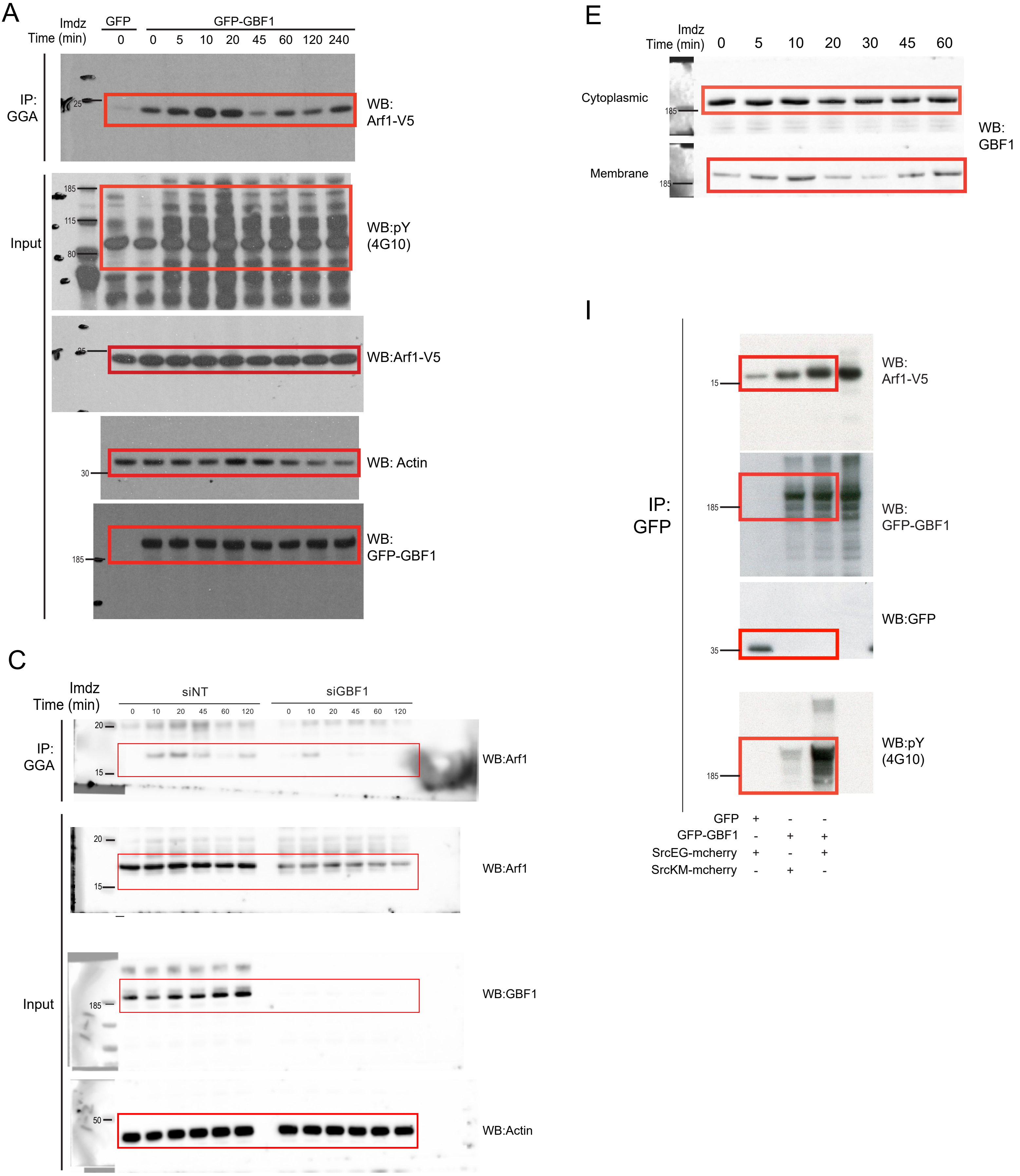
A
Imdz
Time (min)
GFP
GFP-GBF1
0 0 5 10 20 45 60 120 240
IP: GGA
WB: Arf1-V5
Input
WB:pY (4G10)
WB:Arf1-V5
WB: Actin
WB: GFP-GBF1
185
115
80
25
30
E
Imdz
Time (min) 0 5 10 20 30 45 60
Cytoplasmic
Membrane
WB: GBF1
I
IP: GFP
WB: Arf1-V5
WB: GFP-GBF1
WB:GFP
WB:pY (4G10)
15
185
35
GFP + - -
GFP-GBF1 - + +
SrcEG-mcherry + - +
SrcKM-mcherry - + -
C
Imdz
Time (min)
siNT
siGBF1
0 10 20 45 60 120
0 10 20 45 60 120
IP: GGA
WB:Arf1
Input
WB:Arf1
WB:GBF1
WB:Actin
20
15
50

Figure 3

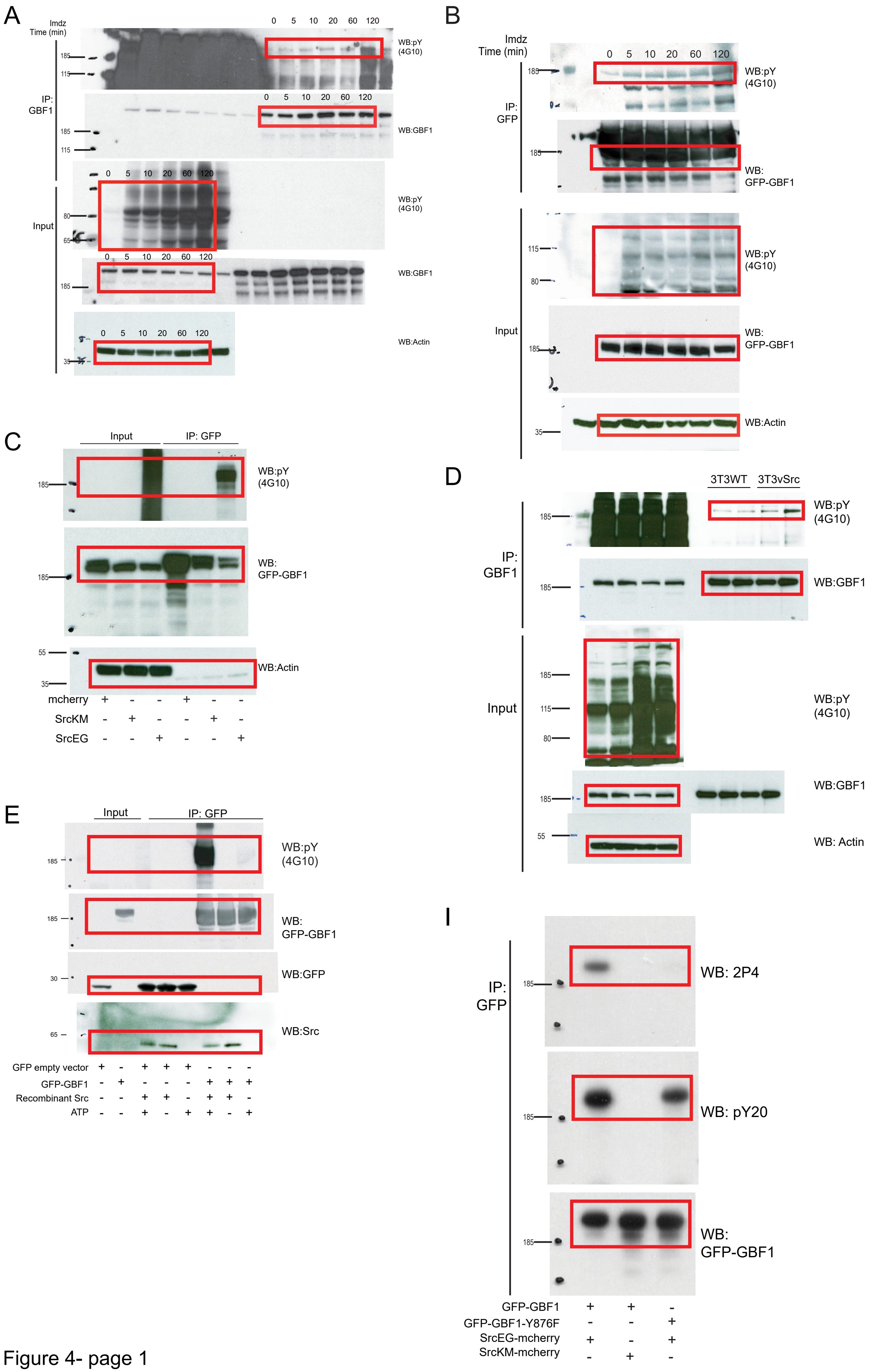

Figure 4- page 1

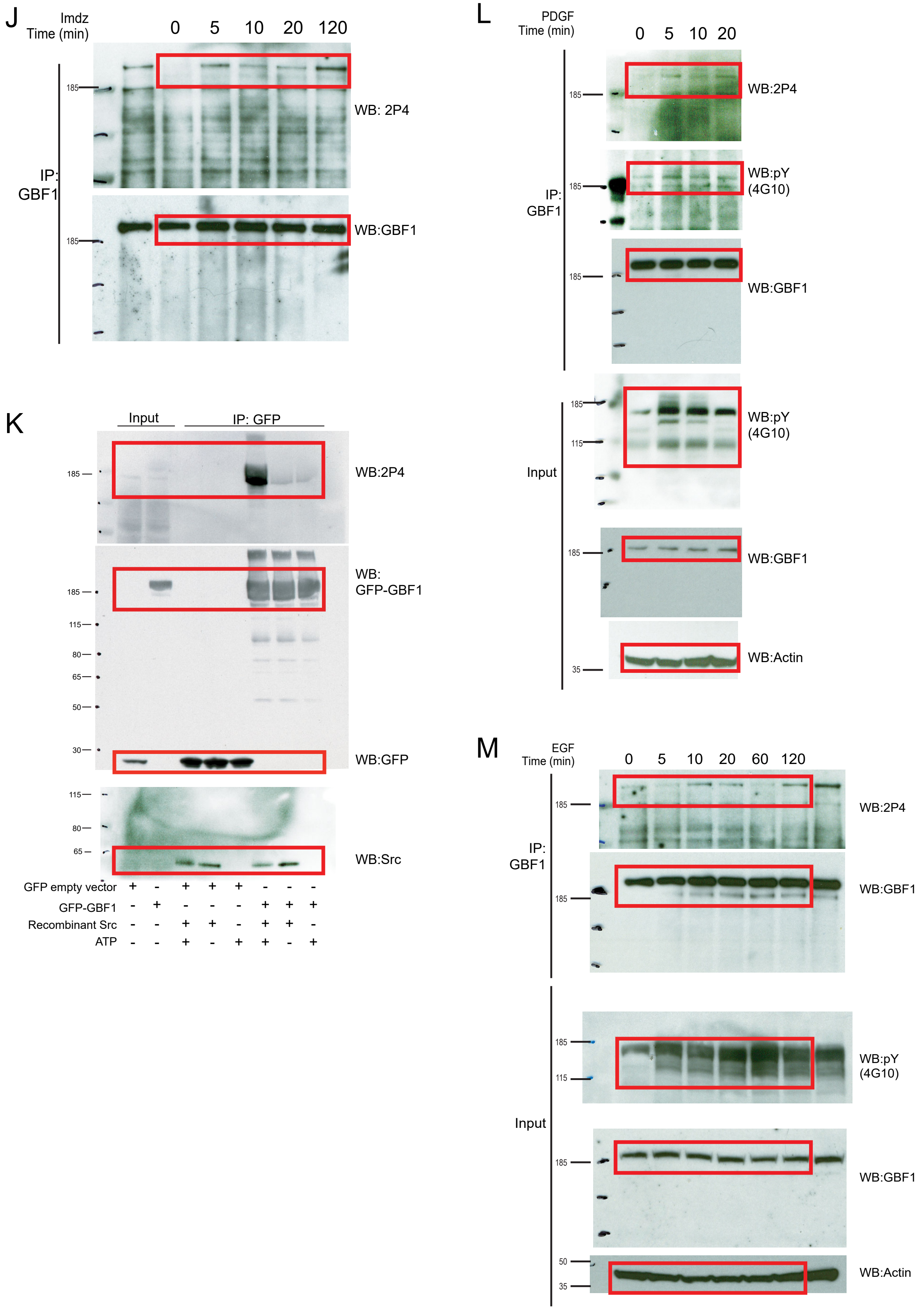

Figure 4-page2

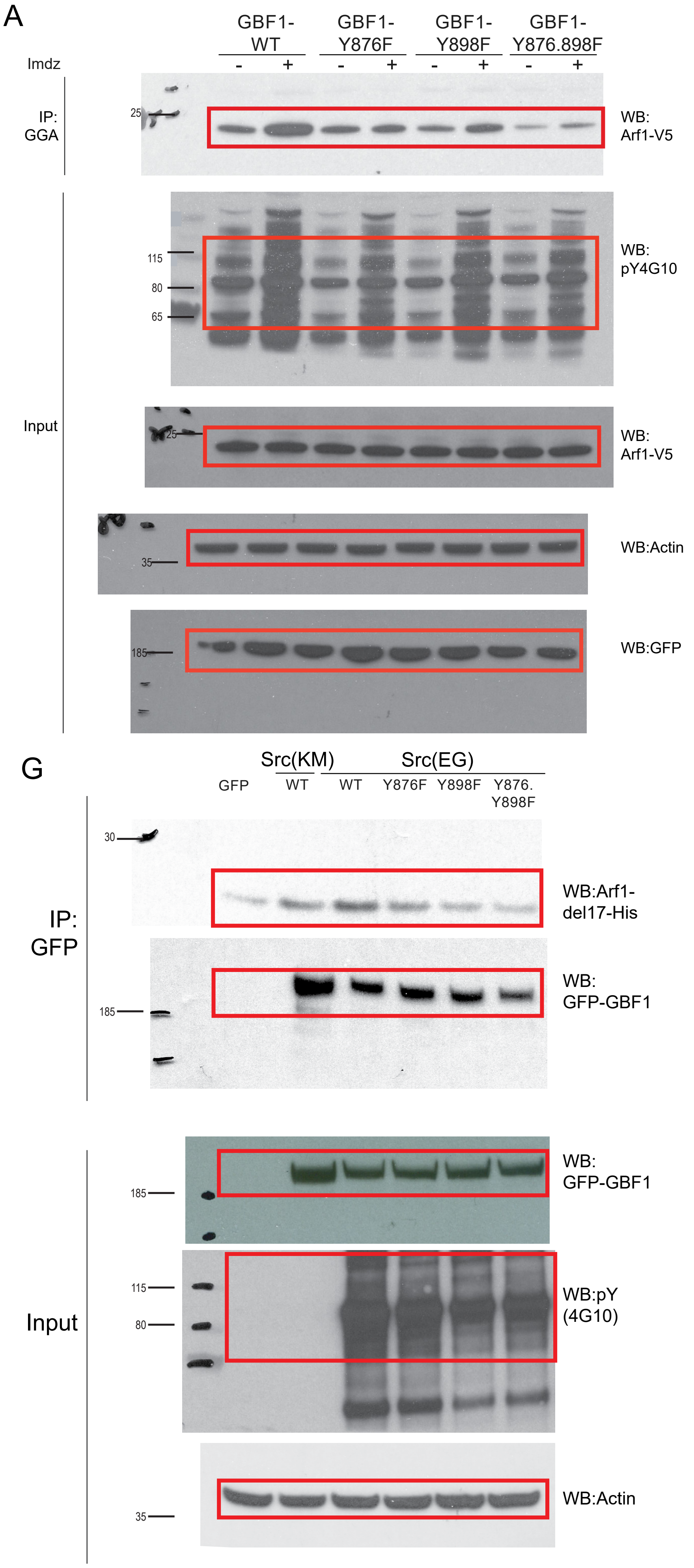
A
GBF1-WT
GBF1-Y876F
GBF1-Y898F
GBF1-Y876.898F
Imdz
- + - + - + - +
IP: GGA
25
WB: Arf1-V5
115
80
65
WB: pY4G10
Input
25
WB: Arf1-V5
35
WB:Actin
185
WB:GFP
G
Src(KM)
Src(EG)
GFP
WT
WT
Y876F
Y898F
Y876.Y898F
IP: GFP
30
WB:Arf1-del17-His
185
WB: GFP-GBF1
Input
185
WB: GFP-GBF1
115
80
WB:pY (4G10)
35
WB:Actin

Figure 5

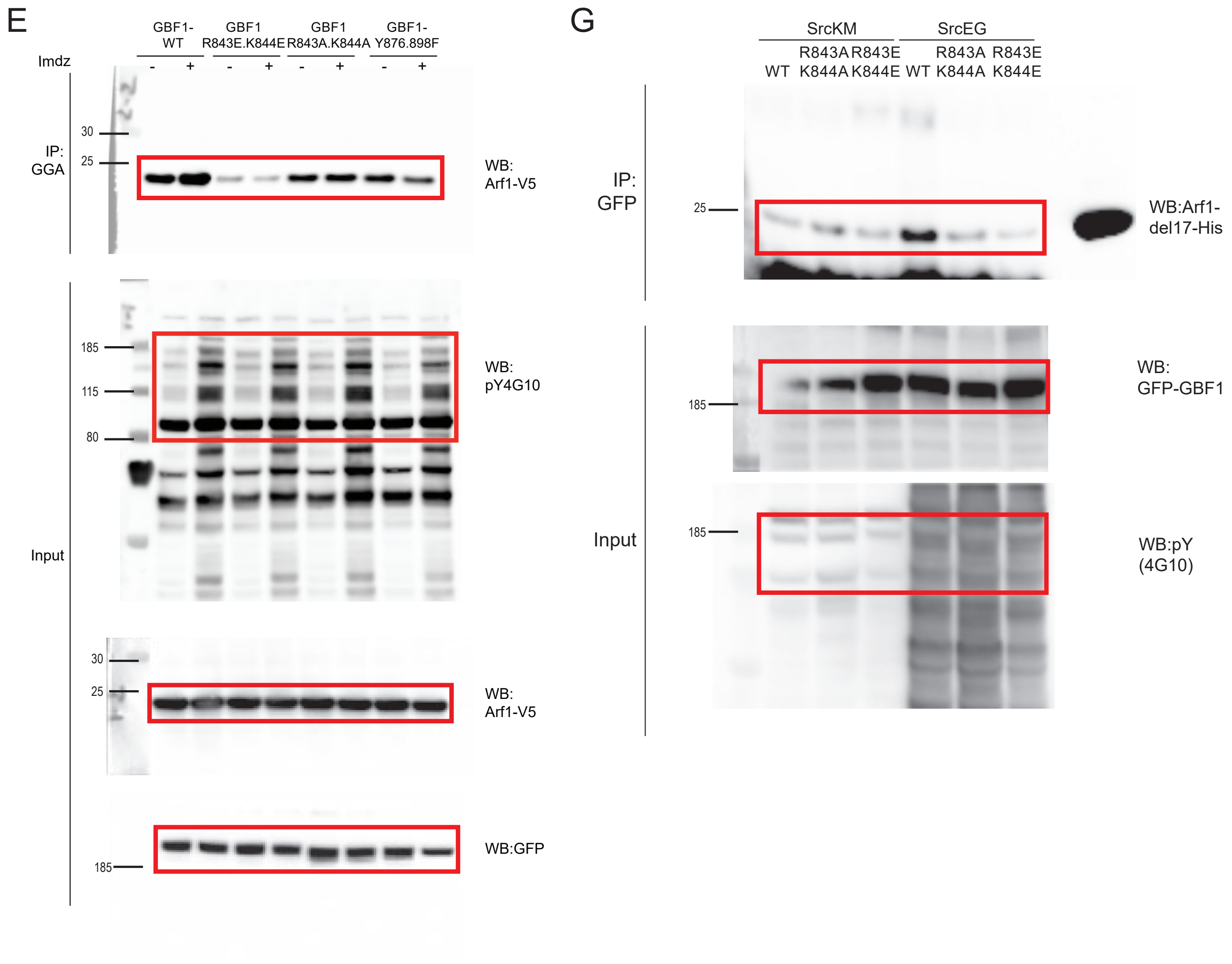
E
GBF1-WT
GBF1 R843E.K844E
GBF1 R843A.K844A
GBF1-Y876.898F
Imdz
- + - + - + - +
IP: GGA
30
25
WB: Arf1-V5
185
115
80
WB: pY4G10
Input
30
25
WB: Arf1-V5
185
WB:GFP
G
SrcKM
SrcEG
WT R843A K844A R843E K844E WT R843A K844A R843E K844E
IP: GFP
25
WB:Arf1-del17-His
185
WB: GFP-GBF1
Input
185
WB:pY (4G10)

Figure 6

B

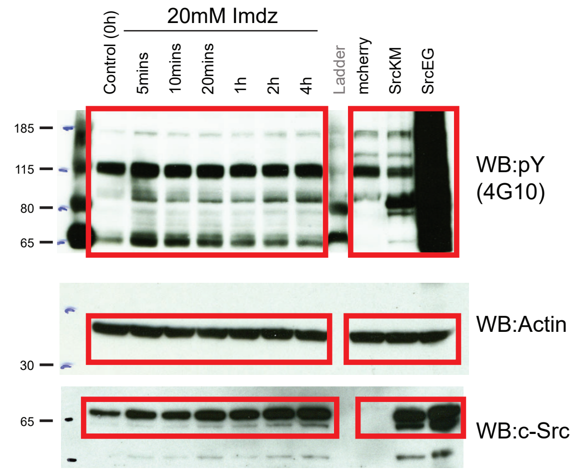

Figure 1- Supplement 1

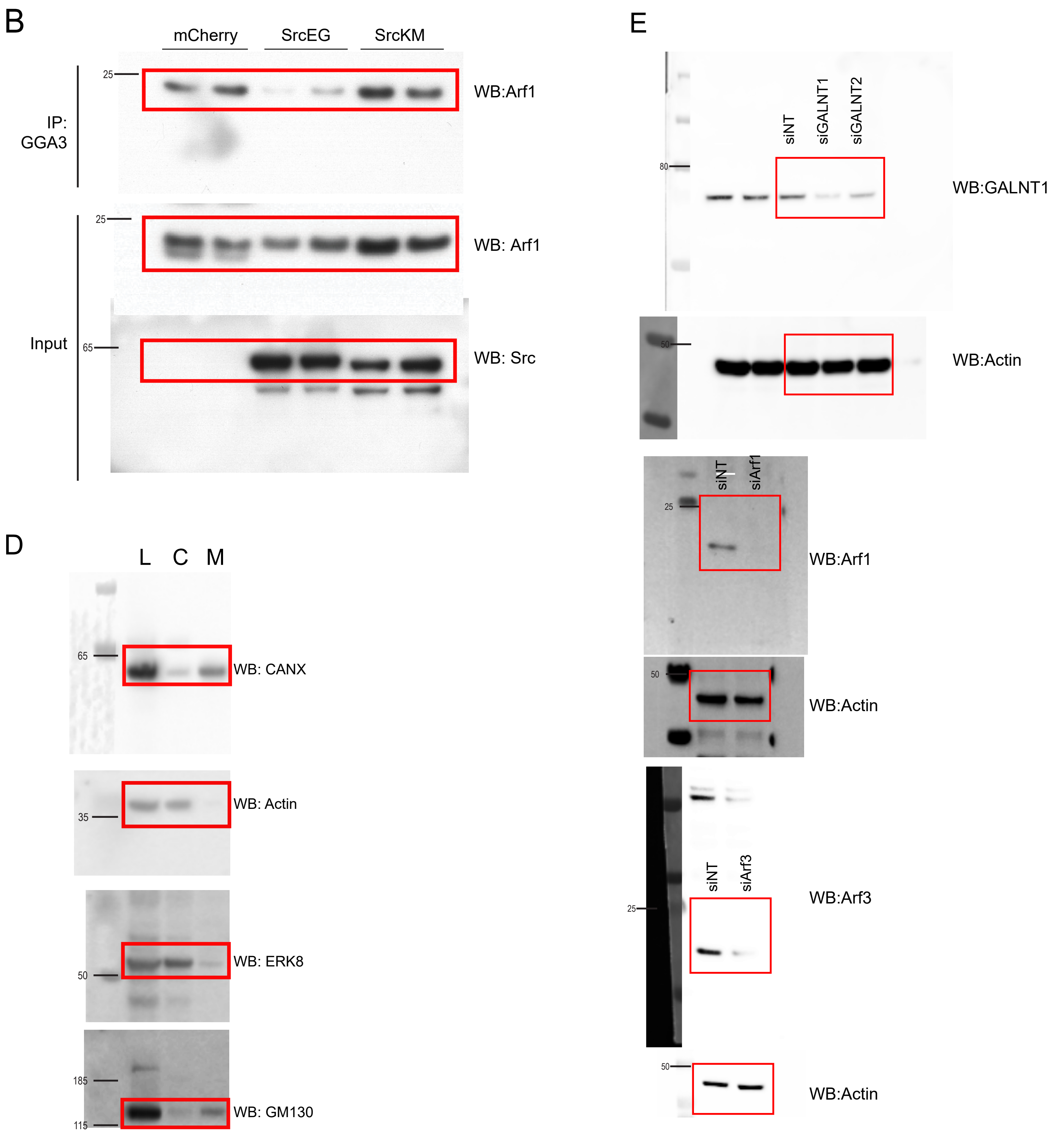

Figure 2- Supplement 1

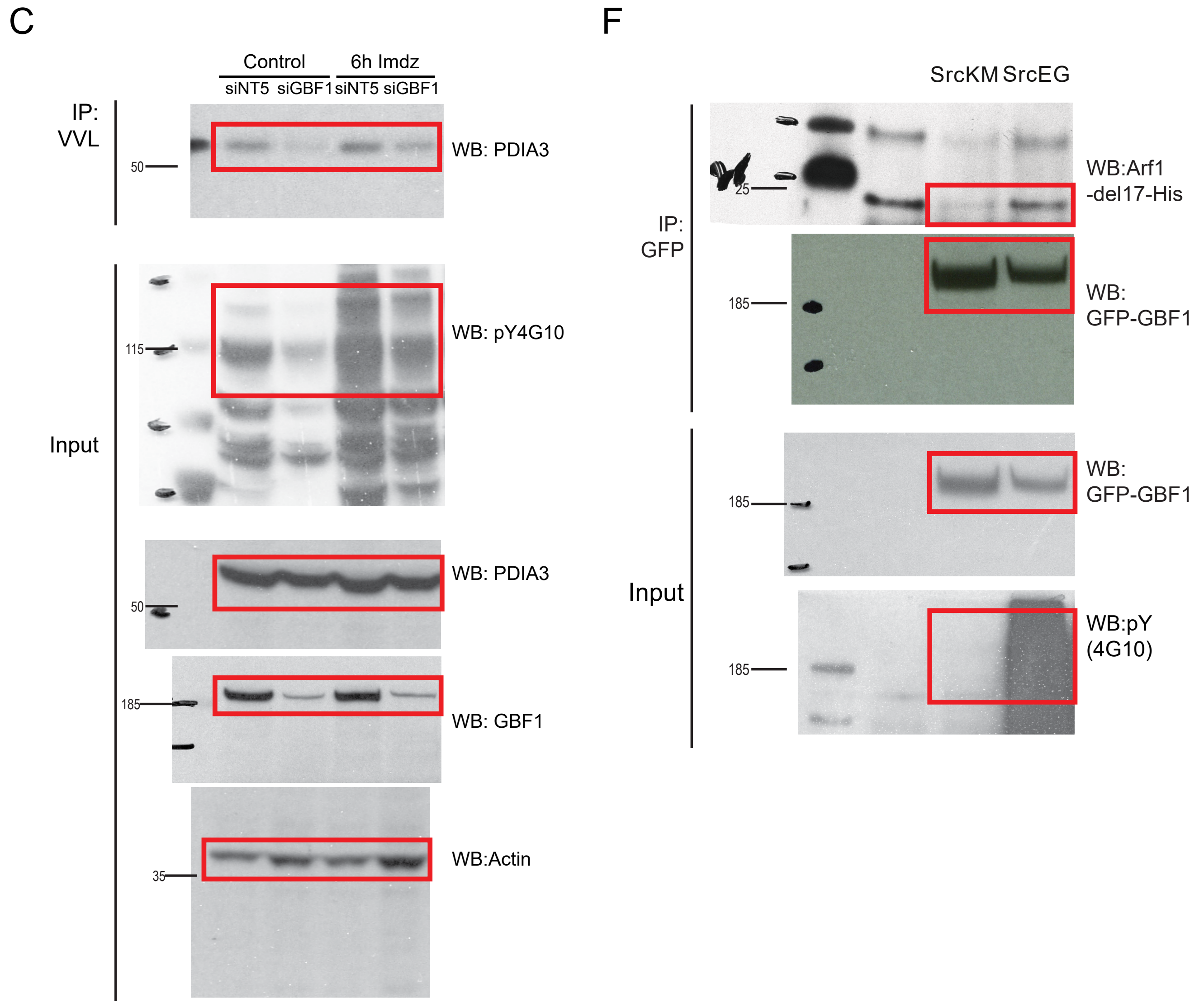

Figure 3- Supplement 1

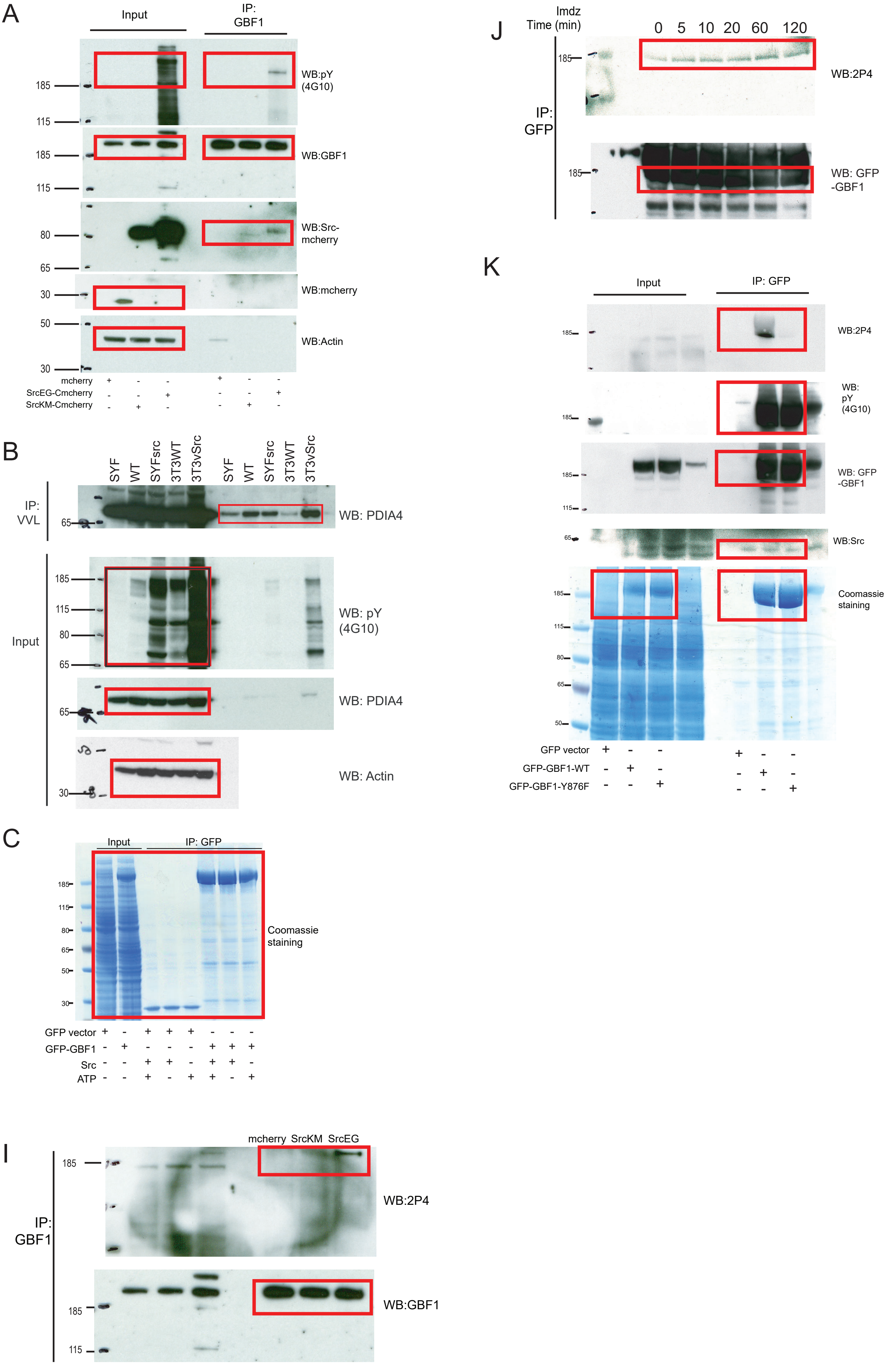

Figure 4- Supplement 1

A

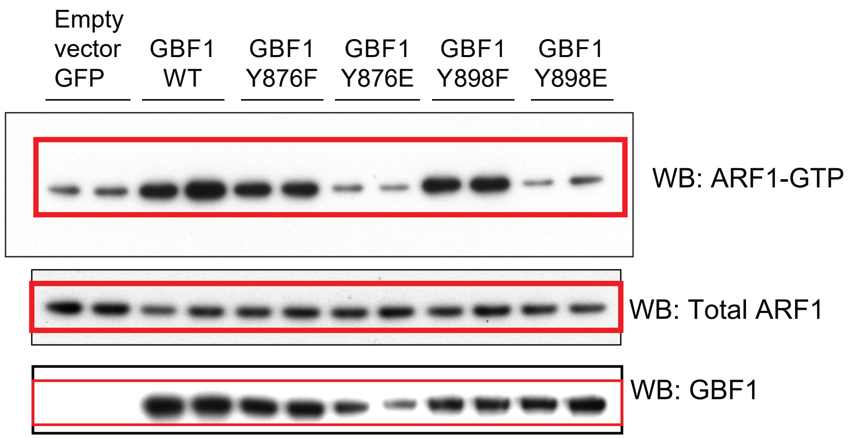
Empty vector GFP
GBF1 WT
GBF1 Y876F
GBF1 Y876E
GBF1 Y898F
GBF1 Y898E
WB: ARF1-GTP
WB: Total ARF1
WB: GBF1
